# Probabilistic Modeling of Chloride Penetration with Respect to Concrete Heterogeneity and Epoxy-Coating on the Reinforcement

**DOI:** 10.3390/ma12244068

**Published:** 2019-12-06

**Authors:** Tuan Duc Le, Petr Lehner, Petr Konečný

**Affiliations:** 1 Faculty of Civil Engineering, Saigon Technology University, 180 Cao Lo Str., Ward 4, Dist. 8, Ho Chi Minh City 73018, Vietnam; tuanld8888@gmail.com; 2 Faculty of Civil Engineering, Department of Structural Mechanics, VSB-Technical University of Ostrava, Ludvíka Podéště 1875/17, 708 33 Ostrava-Poruba, Czech Republic; petr.konecny@vsb.cz

**Keywords:** chloride penetration, concrete heterogeneity, random fields, 2D diffusion model, coatings, reinforcement protection

## Abstract

The presented article demonstrates the probabilistic method based modeling of the 2D chloride ingress into reinforced concrete structures with respect to concrete heterogeneity and epoxy-coated steel reinforcement. Spatial change of concrete diffusion is assessed through the investigation of random variation of the ability of concrete to resist chloride ingress. Time-dependent chloride concentration at the reinforcement level in both homogeneous and heterogeneous models is comparatively considered taking into account of the influence of reinforcement protection as well as the defects and holidays of the coating. Expansion optimal linear estimation method is exploited to generate a random field for the structure at the mesoscale and correlation length is employed to simplify the modeling process. Preliminary analyses of the built model are conducted in both deterministic and probabilistic solutions under the scheme of the finite element method. Thus, possibility of such analyses is exploited.

## 1. Introduction

Durability of concrete exposed to aggressive agents has been a worldwide research topic in recent decades [1,2,3,4,5]. Such agents are chloride ions that reduce the long-term durability of reinforced concrete (RC) structures during their service lifetime. As a result, together with laboratory experiments, modeling of chloride penetration into RC structures has been intensively developed with respect to assessment of the performance of these structures. The fact that parameters governing the durability of RC structures are variable has led to the need for using the probability approach based modeling in simulation works [6,7,8,9]. With the help of such a modeling method, variability of those parameters was included.

In fact, chloride penetration into RC structures is usually modeled under the scheme of homogeneous material in a large scale. A typical example of this common practice can be seen in the work of Lehner et al. (2014) [10] on which the transport of chloride ions through a RC bridge deck was modeled in 2D in order to calculate chloride concentration at the reinforcement level while transverse holidays were assumed. The model was then further developed with consideration of the effect of random scatter of input parameters [11]. At a smaller scale, however, concrete is heterogeneous in nature due to its composite nature. It should be acknowledged also that the induced irregularities in the processes of concrete casting and mixing contributed to the variation. RC modeling at this scale provides a more complex description of the material behavior where effects of concrete aggregate and matrix are investigated under a mesoscale description. The classical lattice model [12] is one of those earliest models although typically applied in fracture mechanics as employed in case of elasto-brittle 1D elements with lattice structure. In that model, elements were constituted from three phases: aggregate, matrix and interface. Due to its characteristic, the model is rather highly demanding with respect to computation and resolution and hence it can be used only for small test specimens. Such a lattice model was later enhanced by 3D rotations of particles and inter-particle contacts were added [13,14,15]. The model was then developed with taking into account of the random fluctuation of mechanical properties of material partly due to random properties of concrete components [16,17].

Dealing with measurement of chloride penetration, typically, it is important to note that the 1D profile is usually a common practice [1,18]. In order to facilitate fast and reliable analysis of heterogeneous concrete, however, new and enhanced measurement techniques were called for. One of such techniques is the laser-induced breakdown spectroscopy system (LIBS). Another promising method is X-ray fluorescence (XRF) [19]. For its application in the field of concrete, see e.g., [20]. The use of this technique offered spatial resolved chloride distributions and quantified ingress profiles regarding to the cement [21]. LIBS was then significantly developed by the Federal Institute for Materials Research and Testing (Germany) with the introduction of a mobile LIBS [22]. When advanced scanning techniques are available, it is suitable to have simulation techniques to mimic physical experiments and hence to prepare models that might reproduce the experimental results faster and with much lower expenses.

As mentioned earlier, it is desirable to build a model that would allow a chloride profile reflecting concrete heterogeneity by which spatial variation of RC material is explored at the mesoscale. Such a model is represented in case of chloride ingress in a work of Jing et al. (2007) [23]. Enhancement in the probabilistic aspect of the work with material description via random field [24] would add a new level of analysis comparing, e.g., to a well-built yet deterministic model [23]. Even though the description of the spatial variability is tested in Bažant et al. (2007) [25], it deserves further evaluation. This model considered the aggregates as spaces with zero penetrability and diffusion was considered to occur only in cement matrix. Another concerned example can be found in Roubin et al. (2015) [26] on which excursion sets of random fields were employed to a morphological description of concrete at mesoscale modeling. In practice, the application of random field regarding penetration of chloride has been challenged by the complication of measurement of random entities due to their fluctuations. Thus, to deal with the modeling of the fluctuations of certain variable parameters, stationary random field was exploited [27,28,29]. To account for, furthermore, type of probability distribution, correlation function and inter-dependent relation among these random variables, the model will be extended under a probabilistic scheme. In probability approach based modeling, correlation length, a parameter representing the rate of fluctuation of random variables was introduced and considered as a characteristic length of the random field [25,30]. This helped to simplify the modeling process.

It also should be acknowledged that reinforcement protection is an important factor in concrete models related to chloride ingress. This aspect has been considered in many research works, e.g., [11,31,32] on which reinforcing bars are coated by epoxy resin in order to increase the time of corrosion initiation. Influence of the defects and holidays in such coating to the performance of bridge decks exposed to chlorides were also examined in the research of [11,32].

Since the models [11,32] do not possess the modeling capability necessary to deal with the description of concrete at mesoscale heterogeneity, 2D chloride penetration probabilistic model of RC slab considering concrete heterogeneity is of interest. An earlier stage of the work including its initially preliminary results was previously published [33]. Currently, the extension to probabilistic application along with random field consideration is focused. The prime development at this stage of the research lies on probabilistic analyses with the addition of the influence of an epoxy coating on the reinforcement to the protection against chloride induced corrosion of the reinforcement. In addition, random variation of resisting ability of concrete to chloride penetration with taking into account of the spatial change of material property—concrete diffusion, is evaluated. Random field of the slab was generated using the expansion optimal linear estimation (EOLE) method [24] according to the implementation conducted by [17] of which basic points are centers of mass of elements. The model was studied and evaluated under the scheme of finite element method (FEM) Homogenous solution with heterogeneous with the conventional steel clad as well as epoxy-coated reinforcement are compared. The comparison is made on the deterministic as well as the probabilistic level.

## 2. Probabilistic 2D Model for Chloride Ingress Heterogeneous RC Structures

The penetration rate of chloride into concrete is generally modeled as a function of depth and time based on the 2nd Fick’s Law of Diffusion:

(1) $$\frac{\partial C\left(x,t\right)}{\partial t}=D_{c}\frac{\partial^{2}C\left(x,t\right)}{\partial x^{2}}.$$

*C*(*x*,*t*) = the chloride ion concentration (%) at a distance x from the surface of concrete in time *t*;
*D_c_* = effective diffusion coefficient (m^2^/s), which characterizes the concrete ability to withstand the penetration of chlorides.

It is also important to note that time dependent chloride diffusion coefficient *D*_*c*,*t*_ is changed during the progress of concrete hydration process [34]. As a result, it can be determined as the following formula as proposed by [35]:

(2) $$D_{c,t}=D_{c,28}{\left(\frac{t_{28}}{t}\right)}^{m}.$$

*D*_*c*,28_ = chloride diffusion coefficient (m^2^/s) measured at selected concrete age;
*t*_28_ = age of concrete measured at period of 28 days (years);
*t* = concrete age (years);
*m* = aging factor.

As above mentioned, correlation length is used herein to define spatial variability of material parameters in the expected model. For stationary random fields, one of the most commonly used models for covariance function is Gaussian model (squared exponential) [36]:

(3) $$C\left(h\right)=\sigma^{2}exp\left(-\frac{h^{2}}{l_{c}{}^{2}}\right).$$

*C*(*h*) = covariance function of single variable *h*;
*l_c_* = correlation length;
*h* = single variable $h=\left\|x_{i}-x_{j}\right\|$;
*x_i_*, *x_j_* = location vectors of point *i* and point *j*;
$\sigma^{2}$ = distribution variance.

The second factor on the right side of Equation (3) can be assigned as a correlation coefficient, $\rho_{ij}$, describing the dependence between two arbitrary points of a random field:

(4) $$\rho_{ij}=exp\left[-{\left(\frac{x_{i}-x_{j}}{l_{c}}\right)}^{2}\right].$$

To generate the random field, in this work, Karhunen-Loeve expansion was exploited with spectrally decomposed covariance matrix *C*. In addition, the Latin hypercube sampling (LHS) approach was used for the sampling of the independent standard Gaussian variables *ξ* and the EOLE method was adopted to deal with extremely computationally expensive evaluation of random field.

This method allows random field evaluation on a regular orthogonal grid of nodes with the spacing of the grid that is about one-third of correlation length. Value at several interparticle facets of the model can be derived from values on the grid by using the following relation:

(5) $${\hat{H}}^{c}\left(x\right)=\sum_{k=1}^{K}\frac{\xi_{k}^{c}}{\sqrt{\lambda_{k}}}\psi_{k}^{T}C_{xg}$$

${\hat{H}}^{c}\left(x_{i}\right)$ = the *c*^th^ realization of the random field ${\hat{H}}^{c}$;
*K* = number of eigenmodes summed up;
*λ* = eigenvalues of covariance matrix of the grid nodes;
*ψ* = eigenvectors of covariance matrix of the grid nodes;
*C_xg_* = covariance matrix between the grid nodes and the center of the facet located at ***x***.

Due to the independence of the EOLE method with the facet system and its geometry, every realization of the random field generated on a large grid can be used for different geometries of the structures and hence time of computation is saved. In this research, functions and procedures are composed using Matlab to generate random field and to assess chloride ingress.

Furthermore, reinforcement epoxy coating was incorporated into the model in the form of the identification of defects in reinforcement. The reinforcement is considered to be fully protected of with the epoxy coating and chloride concentration affecting the reinforcement is considered as zero expect of the holidays (defects) location. In the case of holidays in the epoxy coating, the concentration affecting reinforcement is considered as the same as in the case of non-protected black bar. The model with the epoxy-coating effect is described in details in [11,32]. Towards this end, a FEM algorithm was exploited to find values of chloride concentration at reinforcement level and holidays in the epoxy coating were created by random simulations.

## 3. Numerical Examples

In this part, a test scenario was set up to evaluate the possibilities of the application of the above mentioned combination of the finite element analysis model in the work of Lehner et al. (2014) [10] complemented with the random field application from [17] and probabilistic approach described in a work of Konečný and Lehner (2017) [32] along with a model of epoxy-coated of the reinforcement. It is necessary to underline that the model in a work of Lehner et al. (2014) [10] was originally built with regular triangular mesh targeted at a 2D chloride ingress profile using the simplified version of the transient finite element and Equation (1).

The object investigated in this example was a RC slab exposed to chloride ingress of which dimensions of the cross section were 1.0 m wide and 0.23 m high. The numerical example would be conducted in both deterministic and probabilistic approaches. In the latter case, for the sake of simplification, only one random field would be created with one realization would be made for each basic point in 10,000 simulations. All input parameters for the analyses are summarized in Table 1.

**Figure 1:**
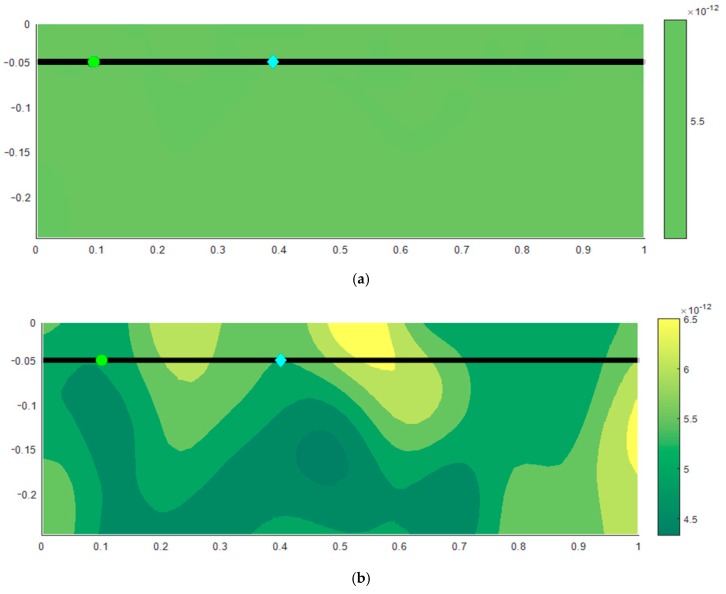
Distribution of the diffusion coefficient in homogeneous model (**a**) including the scheme of the conventional reinforcement (black line) and random field based model (**b**) including the scheme of the epoxy-coated reinforcement (black line) and locations of the holidays in the reinforcement epoxy coating (Epo_1_ = green circle, Epo_2_ = blue rhombus).

The detailed description of input parameters is available in the work of Konečný and Lehner (2017) [32]. The values are constant or their range of values are histograms adopted from literatures [28,32,37,38]. For the definition of spatial variation, the correlation length was considered according to best fit [38] for selected experimental mechanical fracture related data set [28]. The conventional steel reinforcement is compared with the epoxy-coated one. It needs to be noted that in the homogenous solution the results are the same for both approaches. The reason is that the chloride concentration at the reinforcement level is evaluated in the spot with its highest value, but in the case of a homogeneous approach the chloride concentration is the same at the investigated level. The model behaves as a 1D one. If the heterogeneous approach is adopted, then the situation is different. Due to inhomogeneities the concentration of chlorides changes within the reinforcement. Thus, for conventional steel the place with the highest concentration is selected. In case of the epoxy-coated protection the concentration at the holiday level was investigated. Thus, a model called deterministic homogeneous refers both to conventional steel as well as an epoxy coated one. While in the case of the heterogeneous model with other inputs given by deterministic values, the results called heterogeneous refer to conventional steel clad and Epo to a heterogeneous model with an epoxy-coated reinforcement. To consider effects of the epoxy coating for reinforcement protection to chloride concentration at reinforcing bars’ level, in this example, two defects, called holidays, were simulated (Epo_1_ and Epo_2_) as indicated on the Figure 1. It is herein assumed that the problem of mechanical fracture is related to the problem of chloride diffusion because the mesostructure of the concrete composite is of the same nature. Gaussian distribution was also assumed for the diffusion coefficient and the variation coefficient is defined. In this case, the ratio *cv*_S_/*cv*_RV_ = 0.35 between the measured data from cylinders (*cv*_S_ = 0.042) was extrapolated to the random field (*cv*_RV_ = 0.14) [38]. The application of aforementioned ratio into this analysis was a modification of the simulation comparing to that of Le et al. (2018) [33]. It reflected the lower variation of material property in smaller volume of the material such as concrete cylinder comparing to slab or beam cross-section. It is also worth noting that the correlation length, *l*_c_ = 0.1 from the work of Kaděrová (2018) [38] is the value derived for the selected range of slab sizes in fracture bending tests. Thus its application herein was just an analogical consideration and hence this parameter deserves further study attention.

## 4. Results

### 4.1. Deterministic Approach—Distribution of the Diffusion Coefficient

Figure 1 displays the distribution of diffusion coefficient in homogeneous concrete (a) and the heterogeneous one (b). 

It could be observed from the figure that in homogeneous case, the diffusion coefficient was uniform over the studied area while it was ranging from the 4.7 × 10^−12^ to 6.8×10^−12^ m²/s in the heterogeneous case. This scattering of values should better capture the real distribution of different diffusion parameters at the locations of the aggregate, cement components, water, pores and the interspace.

### 4.2. Deterministic Approach—Distribution of Chloride Concentration

**Figure 2:**
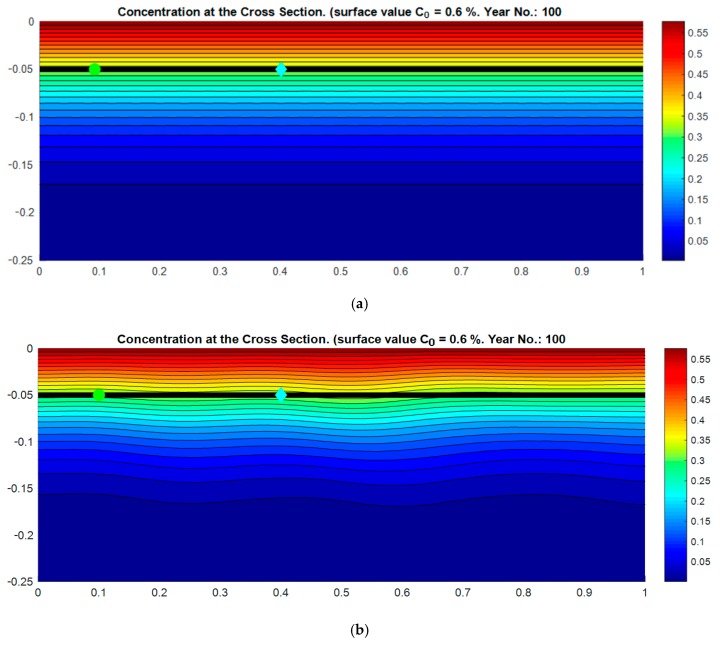
Distribution of chloride concentration in homogeneous model (**a**) at the age of 100 years, including the scheme of the conventional reinforcement (black line) and random field based model (**b**) at the age of 100 years including the scheme of the epoxy-coated reinforcement (black line) and locations of the holidays in the reinforcement epoxy coating (Epo_1_ = green circle, Epo_2_ = blue rhombus).

The resulting chloride ingress 2D profile was computed for considered input parameters and it is given in Figure 2a for homogeneous as well as Figure 2b for heterogeneous models with one random evaluation of random field. 

It can be observed from Figure 2 that the homogeneous case shows uniform penetration over the width of the considered cross-section. The contour lines were straight, and the problem might be calculated as a 1D task giving the same answer for both considered steel reinforcement types.

Thus, the concentration of chlorides at the expected reinforcement is represented by one value. In the heterogeneous case, on the other hand, the contour lines were not straight and the concentration of chlorides showed variation over the length of considered reinforcement.

Thus the concentration of chlorides at the expected reinforcement level might be represented by the scatter of values yielding in differences between the conventional reinforcement and an epoxy-coated one. 

### 4.3. Deterministic Approach—Chloride Concentration over the Time

**Figure 3:**
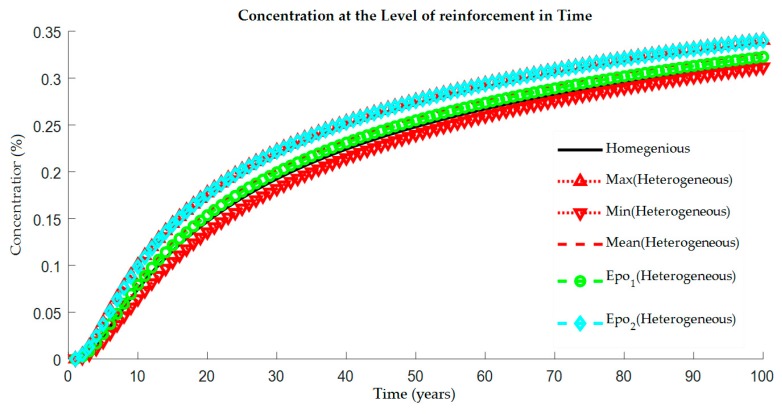
Increase of chloride concentration over the time at reinforcement level for the homogeneous and random field based model for the deterministic solution. The homogeneous model represents both conventional steel reinforcement as well as an epoxy-coated. While the heterogeneous denoted Max, Min and Mean represent the conventional steel. The Epo_1,2_ refer to an epoxy-coated heterogeneous model.

The resulted concentration values were plotted as a function of time in Figure 3. Homogeneous solution had one curve (black colored), whilst the heterogeneous one had three curves for the simulation of the black bar (red colored) and two curves for the simulation of the epoxy coating (green and blue colored).

The variation of concentration values obtained at the reinforcement level was represented by minimum (triangle down), maximum (triangle up) and mean values while the concentration of chlorides in the considered holidays in epoxy-coating protection were described by other two types of marking (Epo_1_ = green circle, Epo_2_ = blue rhombus).

It can be seen that the solution in the case of the heterogeneous model giving us the range of the expected chloride concentration at the reinforcement level. The curve represented the holiday Epo_1_ was very close to the mean values, and on the other hand, the curve represented the holiday Epo_2_ was very close to the maximum values. It is also important to note that the mean values of the heterogeneous model and the results of homogeneous were not same (see Table 2).

In this situation, it could be observed that a better statistical description of the chloride concentration scatter would be obtained with the help of the defined range of standard deviation. Besides, the effect of the holidays in epoxy coating shows that the concentration was affected by the localization of the holidays in reinforcement. Figure 1 revealed that the holiday Epo_1_ was in the area of the higher diffusion coefficient while the holiday Epo_2_ was in the area of the lower diffusion coefficient. Thus, it was expected that the chloride ion concentration values were higher at the localization of the holiday Epo_2_.

### 4.4. Probabilistic Solution

**Figure 4:**
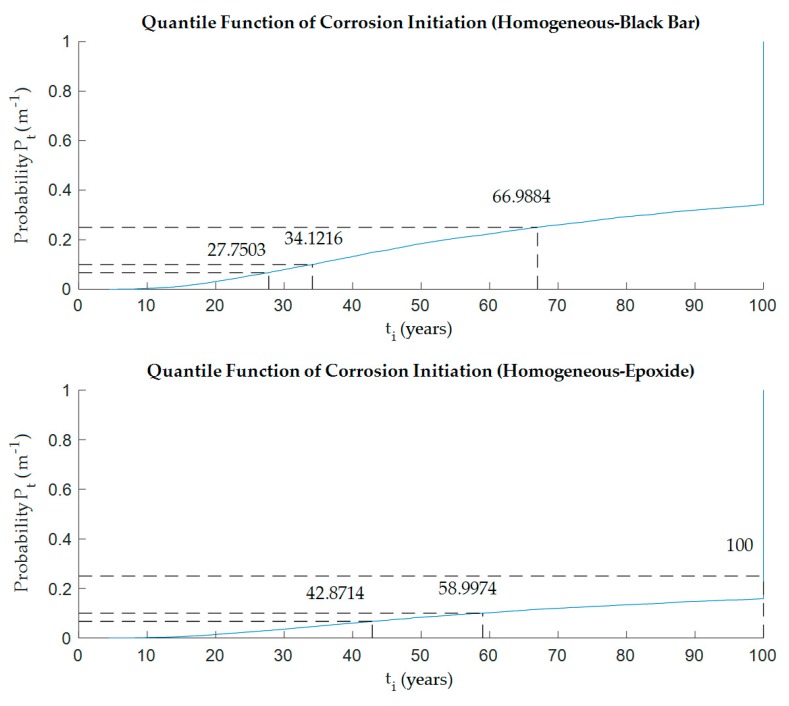
Probability of corrosion initiation, *t*_i_, from the homogeneous model—black bar vs. epoxy-coated reinforcement.

**Figure 5:**
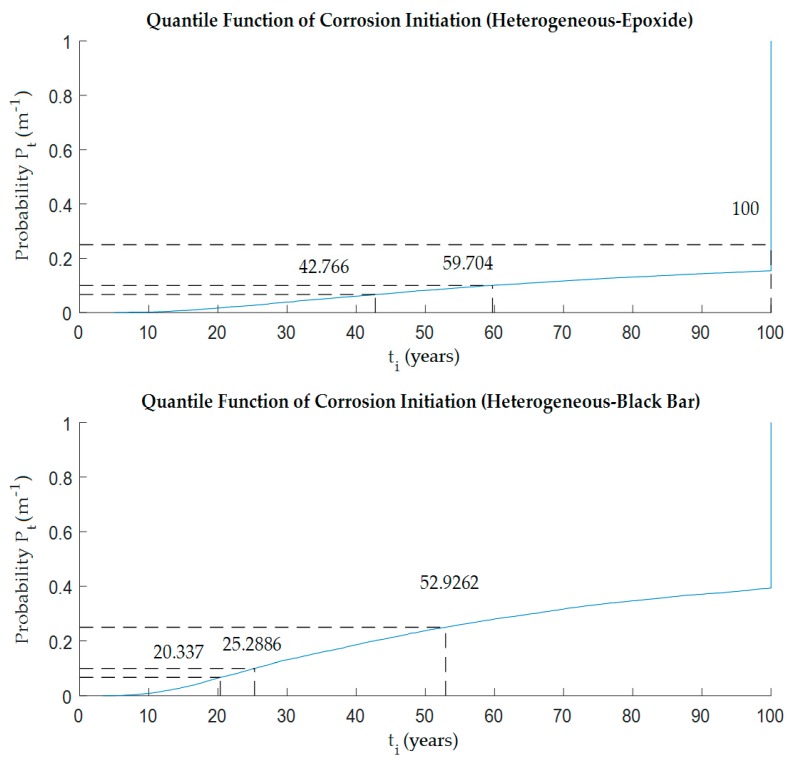
Probability of corrosion initiation, *t*_i_, from the heterogeneous model—black bar vs. epoxy-coated reinforcement.

The FEM model allows repeated analysis of the 2D chloride diffusion problem with input parameters randomly generated by the probabilistic module. Since the deterministic calculation was shown in the previous chapter, as the next step a probabilistic approach was described herein. The Monte Carlo probabilistic solution was applied in each simulation step. Input parameters were set up as random variables according to Table 1. Furthermore, the concentration of chlorides in the evaluated cross-section was calculated for the monitored lifespan of the structure. From each chloride ingress simulation, the highest value of *C*_*xz*,*t*_ at the reinforcement level (black bar) was selected, and this was compared with the *C*_th_ chloride threshold. In the case of epoxy protection of steel reinforcement, the concentration of chlorides was tested simultaneously in locations with protective coating defect. The highest of the chloride concentration values *C*_*xz*,*t*_ was selected for comparison with the *C*_th_ chloride threshold, and a reliability function for the epoxy coating was created. By analyzing the reliability function over time, the time to exceed the limit state was obtained—here the initiation of corrosion. Next, an analysis of the year corresponding to the selected probability of exceeding the chloride threshold and thus initiating corrosion was performed. Probability levels of 5%, 10% and 25% are selected. See Figure 4 for homogeneous model and Figure 5 for heterogeneous model, respectively.

Table 3 summarizes the results from both models. It should be noted that the numerical non-stationary model was used to analyze the first 100 years of life of the structure. If corrosion initiation was not detected, the time to exceed the limit was greater than 100 years.

**Figure 6:**
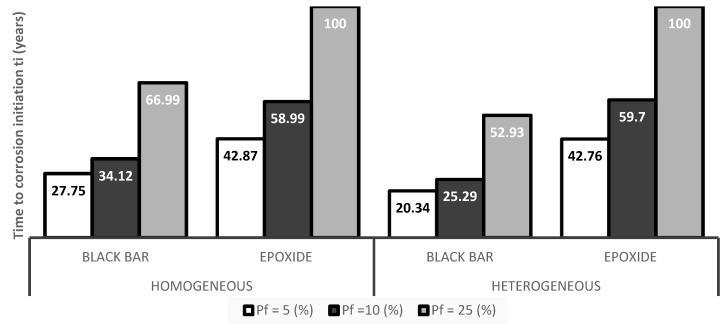
Period to the initiation of corrosion, *t*_i_ (years), for a selected probability level for studied models.

Looking at the probability results (see Table 2 and Figure 6), it could be seen that the possibility of corrosion on the bare reinforcement came much earlier than on the epoxy-coated reinforcement. Regardless of whether it was a homogeneous or heterogeneous model, which was the expected phenomenon. It is very interesting to compare the values for black bar reinforcement and epoxide coating with respect to homogeneity and heterogeneity. It appeared that the type of concrete model did not affect the case with epoxy coating and the values were almost identical for both variants. On the other hand, in the case of the model with conventional steel reinforcement, there were visible differences among the times to corrosion initiation in all probability levels.

It is worth mentioning that in the case of the probabilistic homogeneous solution there was already randomness in the variation of diffusion, however the chloride concentration was the same at all the levels of the reinforcement. Thus the results of the model with an epoxy-coated protection strategy would be the same as in the case of black bar protection, with the exception of the random case where no holiday occurred in the epoxy, and the epoxy was considered to fully protect the reinforcement. The idea of the distribution of chloride concentration was illustrated on the deterministic case on Figure 2a. 

In the case with the application of the random field, the chloride ion distribution illustrated on Figure 2b changed at the reinforcement level due to the spatial change of the diffusion coefficient property (see on Figure 1b). Thus the spatial variability covered in heterogeneous case, and black bar protection affected the concentration in the most exposed location of the conventional reinforcement steel. Authors believed that the reason for similar results in case of epoxy coated protection with homogeneous and inhomogeneous approach and random inputs was that there was already randomness in the selection of holiday points where the concentration of chlorides was analyzed and thus the interaction of spatial variation effect was mitigated with the random selection of investigated points. For example, in one simulation the concentration at the holiday spots might be lower, in another higher, and in another the same as in the homogeneous case.

## 5. Conclusions

The 2D probabilistic chloride ingress model of heterogeneous RC structures set up with the combination of random fields and the 2nd Fick’s Law of diffusion under the scheme of FEM in the previous study of the authors was upgraded in this research focusing on the probabilistic solution. The main updated contents were the inclusion of the reinforcement epoxy coating influence into the model and a modification of the variation coefficient of measured data. The epoxy coating and its defects were simulated by using FEM algorithm and random simulations. Thus comparison of conventional steel and an epoxy-coated one was allowed considering heterogeneity and randomization of input parameters.

The updated model was implemented through a numerical example of chloride penetration into a cross-section of a RC slab. Evaluation of the simulated results revealed that the set up model might be used to address the heterogeneity of concrete including the effect of the reinforcement epoxy coating. Heterogeneous characteristics of concrete were indicated as spatial distribution of chloride diffusion coefficient (in Figure 1b) and by non-parallel contour lines of the chloride concentration (in Figure 2b).

With the inclusion of the epoxy coating and its holidays on reinforcing bars in the case of an otherwise deterministic solution, time dependent chloride concentration at reinforcement level of coated bar (Figure 3) displayed a difference in comparison with that of the black bar (without the epoxy coating). The combination of the epoxy coating holidays and the heterogeneous diffusion model had better resistance values (lower chloride concentration) than the combination of standard reinforcement and the homogeneous diffusion model. It could be concluded that the resulted chloride concentration in the deterministic positions of two holidays of the epoxy coating, lay within the range of the minimum and maximum values of the homogeneous model.

The probabilistic model brought minor different results. Combination of the homogeneous and heterogeneous model gave almost identical resulting values in the case of an epoxy-coated scenario. On the other hand, the black bar reinforcement-based model clearly showed that a homogeneous concrete model calculated a longer lifetime comparing to heterogeneous one. The difference was approximately 30% at all probability levels.

Considering the above mentioned, it could be concluded that if the black bar steel was modeled the heterogeneity should be accompanied with the probabilistic solution of other input parameters. If the epoxy-coated scenario was considered, only the probabilistic solution was enough, hence the need of spatial variation and very high computational burden related to spatial variation of the diffusion coefficient was eliminated.

The meso-structure diffusion model that could be employed for the comparison of the numerical model with the results of LIBS 2D images was also not achieved in this work because the authors did not have access to such technology, and it was planned as a step forward in modeling of chloride penetration.

## Figures and Tables

**Table 1:** Input data for the analyses.

Parameter	Unit	Deterministic Approach	Probabilistic Approach	
			Range/Value	Probability Density Function
Diffusion coefficient, *D*_*c*,28_	× 10^−12^ m²/s	5.585	4.49 ÷ 6.67	Constant [37]
Aging factor, *m*	–	0.284	0.284	Constant [37]
Variation coefficient, *cv_RV_*	–	0.14	0.14	Constant [38]
Width of investigated cross-section, *b*	m	1.0	1.0	Constant [32]
Height of investigated cross-section surface, *h*	m	0.23	0.23	Constant [32]
Size of finite element	m	0.02 × 0.01	0.02 × 0.01	Constant
Depth of reinforcement, *x*	m	0.05	0.04 ÷ 0.11	Histogram [39]
Chloride threshold for corrosion initiation, *C*_th_	% weight of cement	0.2	0.09 ÷ 0.51	Histogram [40]
Concentration of chloride at the surface, *C*_0_	% weight of cement	0.6	0.21 ÷ 1.63	Histogram [32]
Initial concentration of chloride in the cross section, *C*_b_	% weight of cement	0	0	Constant
Frequency of defects in the reinforcement coating, *M*_ashn_	m^-1^	1	0 ÷ 10	Histogram [32]
Relative spacing of the first defect in the reinforcement coating, *M*_ashi_	m	0.2	0 ÷ 1.0	Uniform distribution
Correlation length, *l*_c_	m	0.1	0.1	Constant [28,38]
Monitored life span, *t*	years	100	100	Constant
Number of simulations	–	1	10,000	Constant

**Table 2:** Results of the chloride concentration in 10 years and in 100 years.

Model	Type	10 Years	100 Years
Homogeneous	Black bar as well as epoxy-coated bar	0.075	0.316
Heterogeneous	Maximum (black bar)	0.100	0.340
	Minimum (black bar)	0.064	0.312
	Mean (black bar)	0.081	0.326
	Epo_1_	0.079	0.323
	Epo_2_	0.099	0.339

**Table 3:** Period to the initiation of corrosion, *t*_i_ (years), for a selected probability of the initiation of corrosion for selected variants.

Model	Type	Probability of Corrosion Initiation Pf (%)		
		5	10	25
Homogeneous	Black bar	27.75	34.12	66.99
	Epoxide	42.87	58.99	100.00
Heterogeneous	Black bar	20.34	25.29	52.93
	Epoxide	42.76	59.70	100.00
